# Parotid Metastasis of Early-Stage Upper Eyelid Sebaceous Carcinoma: A Case Presentation and a Literature Review

**DOI:** 10.7759/cureus.56838

**Published:** 2024-03-24

**Authors:** Athanasios Ioannidis, Efthymios Kyrodimos, Dimitra Riga, Irineos Nikolopoulos, Aristeidis I Giotakis

**Affiliations:** 1 Otolaryngology, Head and Neck Surgery, Hippocration Hospital, University of Athens, Athens, GRC; 2 Pathology, Hippocration Hospital, University of Athens, Athens, GRC; 3 Otolaryngology, Head and Neck Surgery, Ippokrateio General Hospital of Athens, Athens, GRC

**Keywords:** case report, lymph node metastasis, regional metastasis, parotid, sebaceous carcinoma

## Abstract

Metastasis from early-stage sebaceous carcinoma of the eyelid to the salivary glands is considered very rare, occurring in less than 3% of early-stage patients. We report the case of a 72-year-old Caucasian man with a parotid tumor. Fine needle aspiration was consistent with a salivary duct carcinoma. A subtotal parotidectomy with ipsilateral neck dissection was performed. The pathology report revealed a sebaceous carcinoma with one parotid and two cervical lymph nodes infiltrated. The patient had a history of an early-stage sebaceous carcinoma of the upper eyelid two years before, which was revealed after the histological examination. An early-stage eyelid sebaceous carcinoma can metastasize to lymph nodes of the parotid glands. A close follow-up should not be neglected.

## Introduction

Ocular sebaceous carcinoma arises from the meibomian glands, glands of Zeis, or glands associated with the caruncle [1,2]. In the head and neck, sebaceous carcinoma most commonly occurs in the eyelid [1]. Its incidence is approximately 1-5, 5% of eyelid malignancies in the Caucasian population [1]. Regional lymph node metastasis can occur in up to 44% of advanced cases and less than 3% in the early stages [3,4]. The most common sites of lymphatic regional metastasis are the preauricular, submandibular, and parotid lymph nodes [2]. While parotid lymph nodes are considered a common site of metastasis, the data are scarce on the number and frequency of early-stage tumors [5-7]. 

Here, we report the case of a 72-year-old man with a metachronous metastasis in the parotid gland of a previously resected early-stage sebaceous carcinoma of the eyelid. Furthermore, we provide a literature review. The institutional review board approved this case report (704-09/01/2024).

## Case presentation

**Figure 1:**
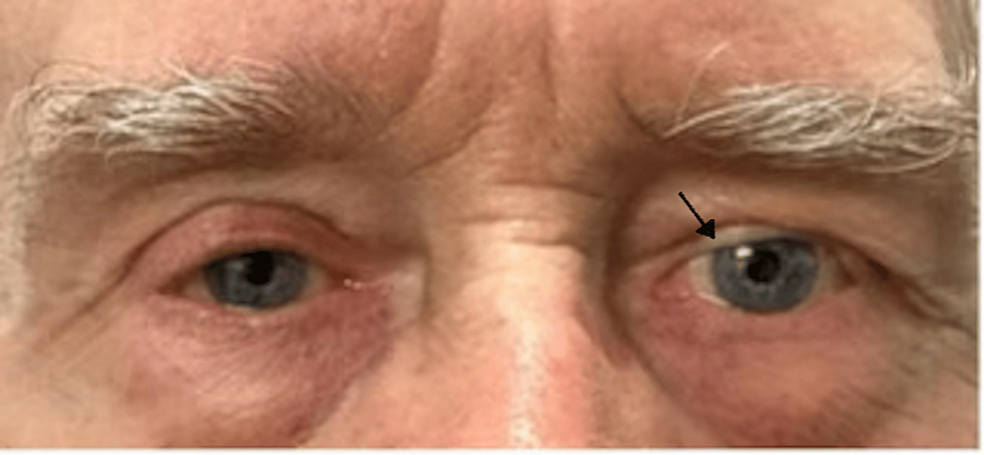
En face picture of the patient. Note the defect of the left upper eyelid marked with an arrow (compared to the right one) after the left upper eyelid blepharoplasty

A 72-year-old Caucasian male presented in the outpatient clinic with a firm, painless, palpable mass of the left parotid gland, which was first noticed by himself six months ago. The patient did not exhibit any other symptoms, had any medical or familial medical background, and did not mention any drugs or allergies. He mentioned a left upper eyelid blepharoplasty due to a vascular anomaly two years ago (Figure 1).

**Figure 2:**
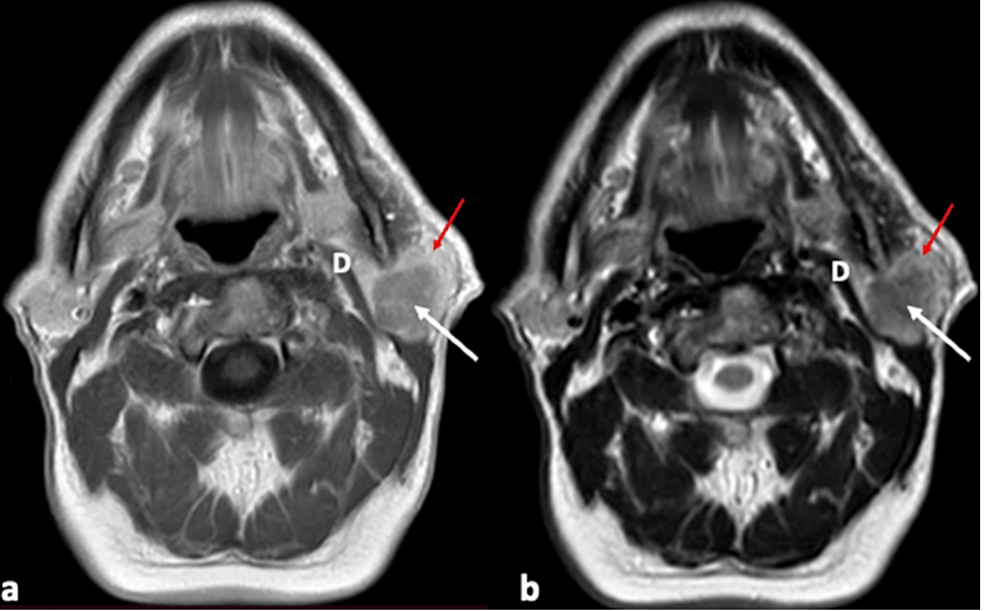
MRI, axial view: (a) T1-weighted and (b) T2-weighted. Parotid gland (red arrow); tumor (white arrow) MRI, magnetic resonance imaging; D, digastric muscle

Physical examination revealed a firm, fixed mass on the left parotid region without palpable cervical lymph nodes. The facial nerve function and the saliva drainage through the Stensen’s duct were normal. No other skin lesions and no involvement of the rest lower cranial nerves were detected. The patient had undergone computed tomography (CT) and magnetic resonance imaging (MRI) that showed a homogenous lesion with irregular borders on the superior lobe of the left parotid gland. The lesion showed low intensity on T1-weighted and intermediate intensity on T2-weighted images. The mass showed uptake of the intravenous contrast on both CT and MRI (Figure 2).

**Figure 3:**
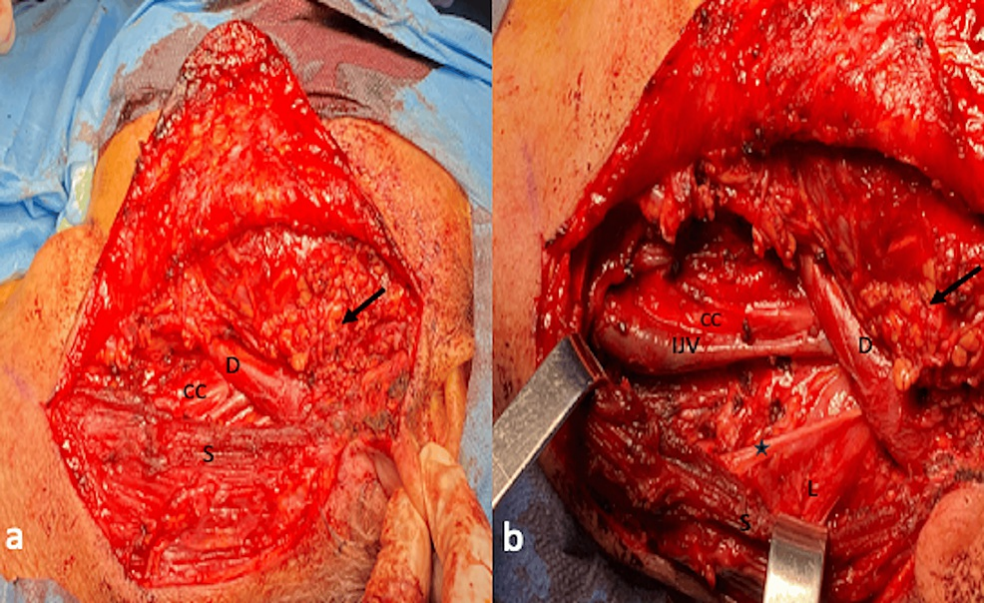
Intraoperative images. (a) Without instruments; (b) after pulling the sternocleidomastoid muscle posteriorly with two farabeuf retractor instruments S, sternocleidomastoid muscle; D, digastric muscle; black arrow, remaining parotid tissue; IJV, internal jugular vein; star, accessory nerve; L, levator scapulae muscle; CC, common carotid artery

**Figure 4:**
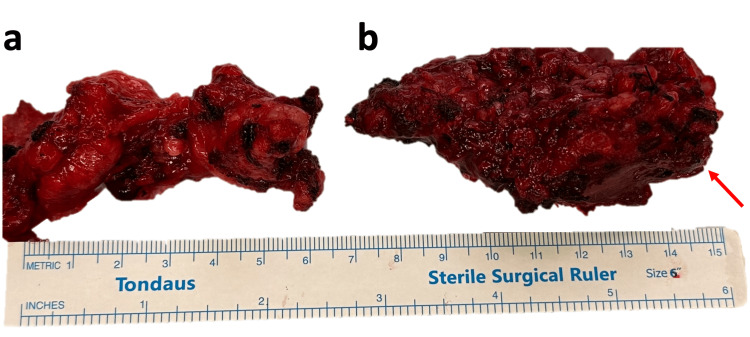
Surgical specimens. (a) Cervical lymph nodes from levels II and III; (b) parotid gland with the tumor (red arrow points to the tumor). The figure’s background was slightly modified to remove blood stains

Cytologic examination of the fine needle aspiration specimen revealed hypercellular smears with plenty of cellular clusters as well as abundant separate neoplastic glandular cells. The neoplastic cells were medium-sized, ovoid, with an eccentric, hyperchromatic nucleus, coarse chromatin, irregular nuclear membrane, with occasionally obvious nucleoli and solid or vesicular cytoplasm, giving the impression of intracellular mucin. They were arranged in flat clusters, or adenoid and papillary formations, advocating the diagnosis of adenocarcinoma, most likely salivary duct carcinoma of the parotid gland. CT of the thorax revealed no pathologic findings. The multidisciplinary tumor board opted for subtotal parotidectomy and ipsilateral selective neck dissection of levels II and III. Through a modified Blair incision with extension to the neck, the parotid gland was mobilized from the sternocleidomastoid and digastric muscle as well as from the auricular cartilage. After identification of the main trunk of the facial nerve, the parotid gland with the tumor was dissected from the facial nerve. The tumor was adjacent to the marginal branch of the facial nerve without infiltrating it. Following confirmation of normal facial nerve function with neuromonitoring, an ipsilateral selective neck dissection of levels II and III was performed and the specimens were sent to the pathology department for histological examination (Figure 3 and Figure 4).

The patient received postoperatively antibiotics and non-steroidal anti-inflammatory drugs. The drain was removed on the third postoperative day. The patient was discharged on the fourth postoperative day in good condition. The pathology report was indicative of metastasis of sebaceous carcinoma in the parotid gland. 

A careful medical history was sought about the exact nature of the vascular anomaly resected from the upper eyelid. The patient provided the histology report, which revealed an R0 resected sebaceous carcinoma. The patient was staged back then as T2aN0M0, according to the 7th edition of AJCC or T1bN0M0 according to the 8th edition. The patient reported no further treatment. He has missed his follow-up since then.

Gross inspection revealed a surgical specimen composed of a parotid gland tissue specimen measuring 4.5x4.5x3 cm as well as an adipose tissue specimen measuring 7x5x1 cm, which included 18 lymph nodes, measuring from 0.3 to 1 cm in diameter. The cut section of the parotid gland specimen revealed a poorly circumscribed tan-white and solid lesion measuring 2.8 cm in greatest dimension. 

**Figure 5:**
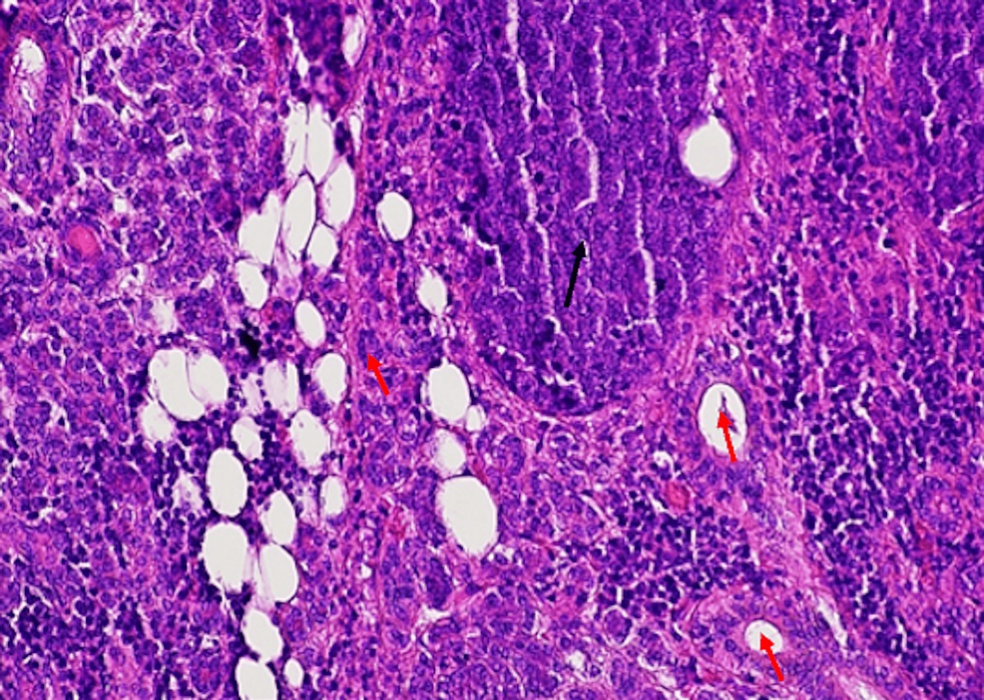
Hematoxylin-eosin stain 10×: invasion of the parotid gland by a malignant epithelial tumor composed of cellular nests and sheets (black arrow). Seromucinous parotid glands (red arrow)

**Figure 6:**
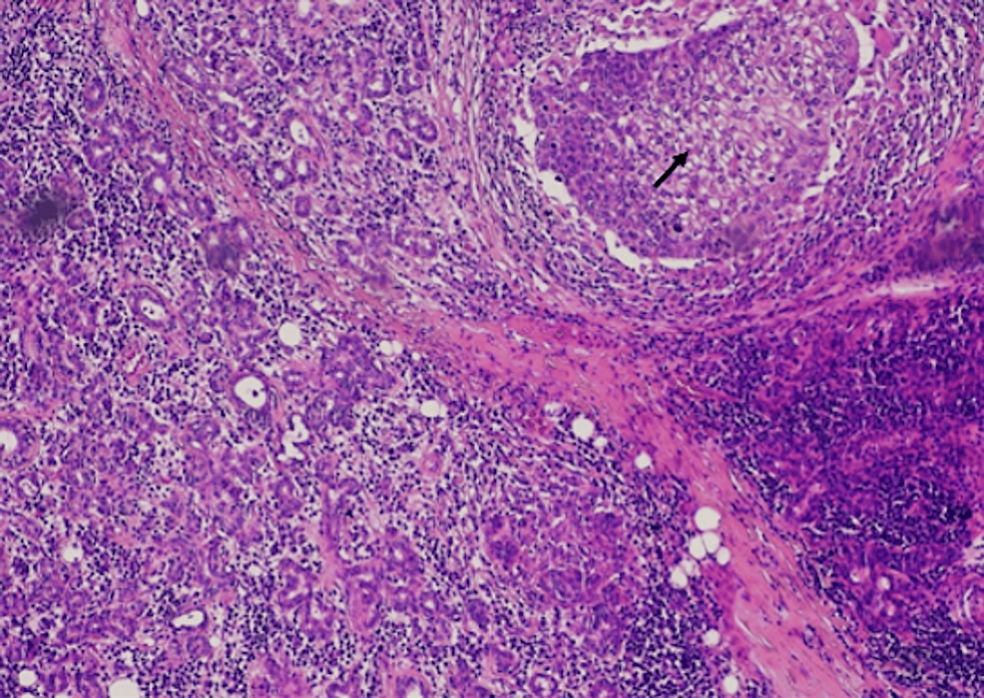
Hematoxylin-eosin stain 10×: tumor nests composed of malignant cells invading seromucinous parotid glands (black arrow)

**Figure 7:**
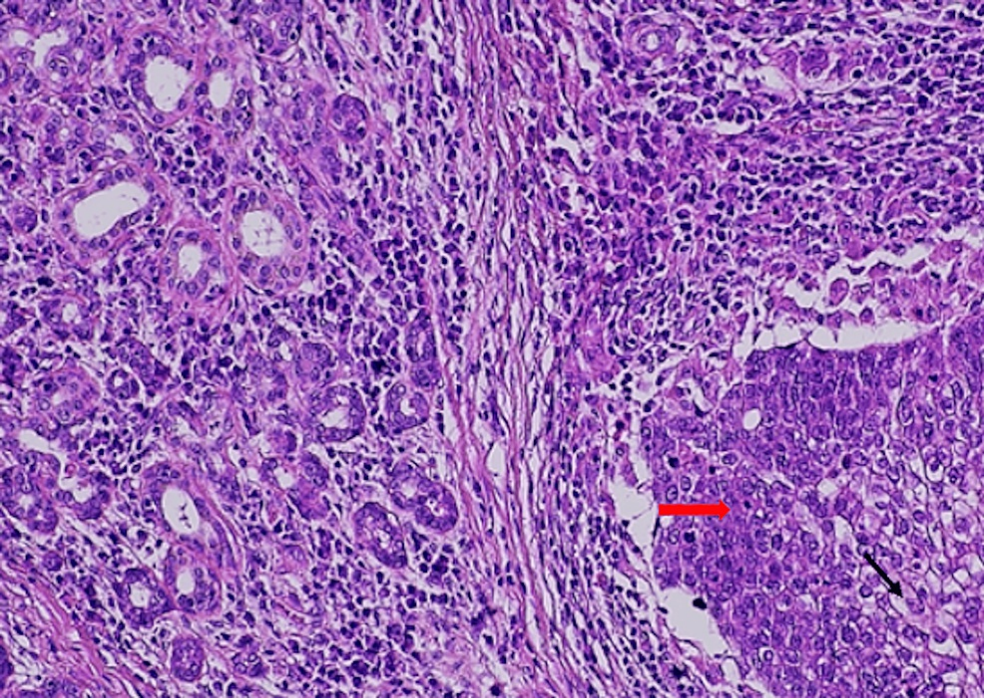
Hematoxylin-eosin stain 20×: solid tumor nest invading the parotid glands. (red arrow) Malignant cells have hyperchromatic nuclei, abundant clear vacuolated to eosinophilic cytoplasm and mild to marked cellular pleomorphism, and marked cytological atypia (black arrow)

Microscopic examination of the lesion revealed a tumor, with multiple, variable-sized nests and sheets of neoplastic cells, without peripheral palisading, composed of tumor cells with hyperchromatic nuclei, abundant clear vacuolated to eosinophilic cytoplasm and mild to marked cellular pleomorphism and marked cytological atypia. Perineural invasion, squamous metaplasia, atypical mitoses, and tumor cell necrosis were also observed (Figures 5-7). Three lymph nodes were positive for metastasis of sebaceous carcinoma. The patient was staged as rT0N2bM0.

**Figure 8:**
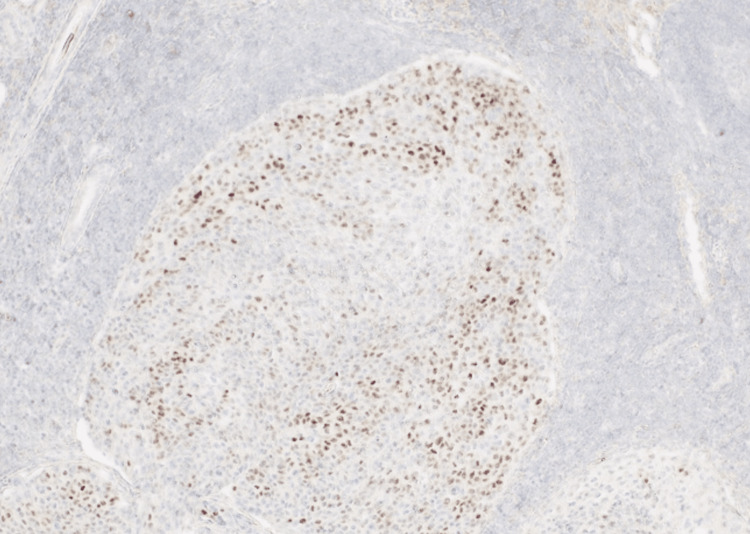
Androgen receptor stain 10×: malignant cell positivity was observed in 30% of the tumor cells. This figure highlights a “hot-spot” area of androgen receptor positivity

**Figure 9:**
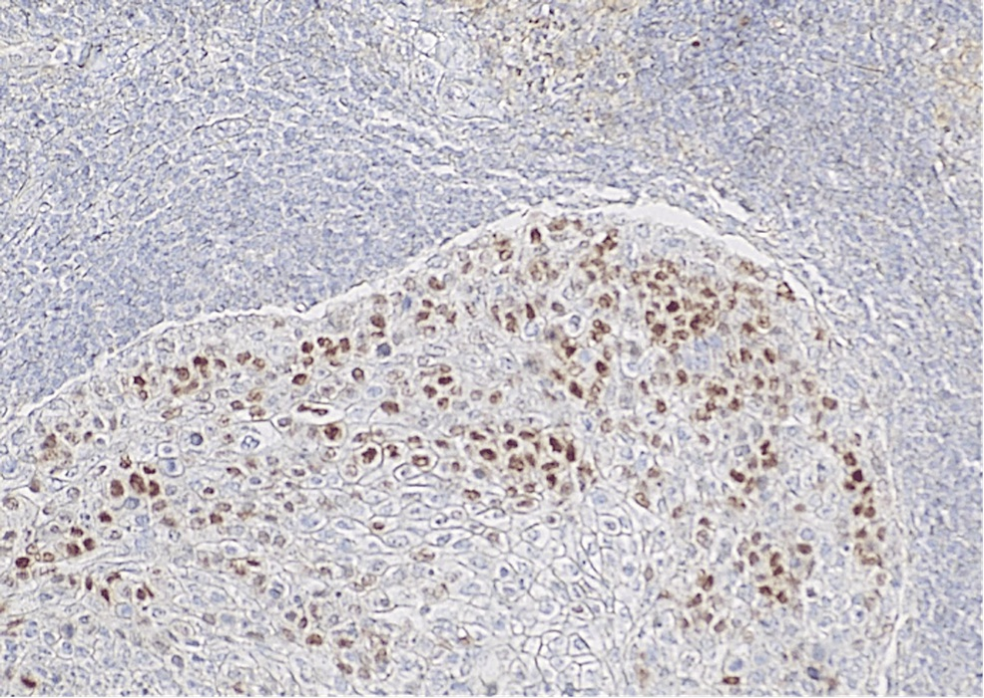
Androgen receptor stain 20×: nuclear expression of androgen receptor antibody

**Figure 10:**
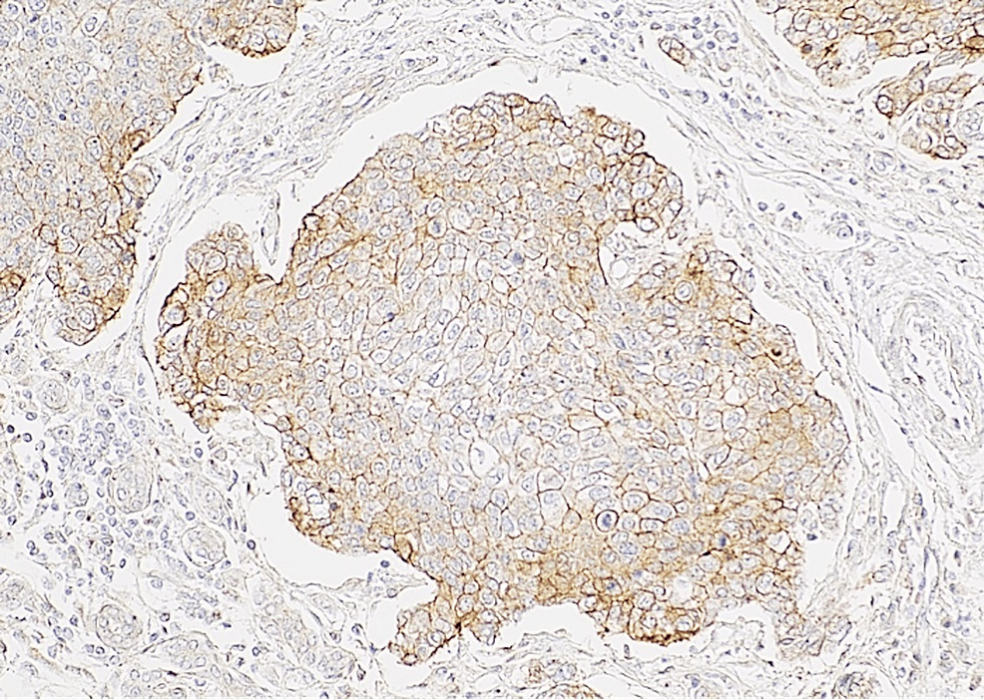
HER2 stain 40×: moderate to strong and complete membranous positivity of malignant cells in HER2 antibody

Immunohistochemical investigation of the tumor included epithelial membrane antigen (EMA), HER-2, androgen receptor, S-100, BerEp4, and P63 antibodies. The immunohistochemical positivity of androgen receptor in 30% of the tumor cells, HER-2 and EMA antibodies in the majority of the neoplastic cells, in conjunction with the absence of reaction for S-100, BerEp4, and P63 antibodies, as well as the clinical history of sebaceous carcinoma of the eyelid, led to the exclusion of squamous cell carcinoma and basal cell carcinoma of the parotid gland (Figures 8-10). We concluded with the diagnosis of metastatic sebaceous adenocarcinoma of the eyelid to the parotid gland and co-excised lymph nodes.

The multidisciplinary tumor board suggested adjuvant chemoradiotherapy. The patient was referred to a radiooncologist. He received 66 Gy on the parotid region, 60 Gy on the high-risk cervical lymph nodes, and 54 Gy on the low-risk nodes. Furthermore, he was placed into a Carboplatin AUC2 regimen for six weeks. Patient status was complete remission on the six-month follow-up.

## Discussion

The case presented highlights the significance of maintaining a structured follow-up regime in all cases, regardless of their stage. Data on the metastasis of early-stage sebaceous eyelid carcinomas are scarce. Such cases are not commonly associated with lymph node metastasis, and diagnosis can be elusive. After confronting such a case, we intended to share our knowledge on this subject, based on proper documentation throughout management and treatment. The aim was also to perform a literature review and check on similar cases that were found with lymph node metastasis. For this, the PubMed database was utilized to identify cases of parotid metastasis of sebaceous carcinoma of the eyelid. The following keywords were used: sebaceous carcinoma AND (parotid metastasis OR regional metastasis). Further search on related articles was performed. Cases of primary sebaceous carcinoma of the parotid were excluded. 

Early-stage sebaceous carcinomas of the T stage lower than T2b or smaller than 10 mm on greater dimensions were not associated with lymph node metastasis [7,8]. There is a lack of adequate data that correlates the stage of the disease with the probability of lymph node metastasis. No initial TNM stage was documented in most reported cases of patients with sebaceous carcinoma of the eyelid and regional metastasis. There were only seven studies listing the cases of ocular sebaceous carcinoma with regional metastasis in correlation to the TNM stage (Table 1). These studies were referring to regional metastasis rather than parotid specifically, which was, however, stated as a very common site of regional metastasis [5,9-14]. The current study focuses mostly on early-stage patients with regional metastasis.

**Table 1:** Studies reporting regional metastasis of early-stage ocular sebaceous carcinoma ^*^7th TNM edition ^#^8th TNM edition ^~^Total cases ^+^Early-stage ocular sebaceous carcinoma ^^^Regional metastasis ^>^Choi et al. reported 40 cases of ocular sebaceous carcinoma. One patient presented with a T1 tumor, 20 patients with a T2a tumor, and 11 with a T2b tumor. The authors noted regional metastasis in two patients with a T2a tumor and two patients with a T2b tumor

Data	Total^~^	ES^+^					RM^^^						
		T1	Τ2	T2a	T2b	T2c		T1	Τ2		T2a	T2b	T2c
Watanabe et al.* [10]	63	0	NA	38	0	0		0	NA		2	2	0
Kaliki et al.* [11]	191	1	111	NA	NA	NA		0	9		NA	NA	NA
Esmaeli et al.* [5]	50	7	NA	4	25	0		0	NA		0	3	0
Sa et al.^# ^[12]	100	30	NA	0	13	8		1	NA		0	1	1
Lam et al* [13]	22	0	NA	16	3	0		0	NA		0	1	0
Choi et al.* [14]	40	1	NA	20	11	0		0	NA		2	2^>^	0
Nasser et al.* [15]	65	6	NA	11	19	0		0	NA		0	2	0
Current study*	1	0	NA	1	0	0		0	NA		1	0	0

Watanabe et al. reported two cases of T2a with regional metastasis on their cases series that were measured under 10 mm on greater dimensions, stating that tumors <10 mm in size were associated with regional nodal metastases [9]. Tryggvason et al. collected data from the SEER database and reported extraocular head and neck, as well as ocular sebaceous carcinomas. The authors found four cases with tumors smaller than 10 mm with regional metastasis and stated that ocular sebaceous carcinomas had a higher incidence of nodal metastasis. However, they did not define which of these four cases were of ocular origin [15]. Sa et al. found in their review one case of T1 with regional metastasis on the follow-up [11]. Choi et al. reviewed 40 patients treated at Seoul for sebaceous carcinoma of the eyelid and found among them two cases staged as T2a with regional metastasis [13]. All the studies that investigated the probability of metastasis of sebaceous carcinoma according to the TNM stage suggested a sentinel lymph node biopsy for stages higher than T2b or T2c tumors [9-15].

The above-mentioned data implied that only five other patients with ocular sebaceous carcinomas measuring under 10 mm or of a lower than T2b stage (7th AJCC) have shown regional metastasis. To the best of our knowledge, this is the sixth case reported, with proper documentation throughout its management. Our case highlighted the significance of a structured follow-up.

## Conclusions

Early-stage eyelid sebaceous carcinomas might metastasize to the lymph nodes of the parotid gland. Close follow-up should not be neglected in all patients.
